# Population-Level Effect of Cholera Vaccine on Displaced Populations, South Sudan, 2014

**DOI:** 10.3201/eid2206.151592

**Published:** 2016-06

**Authors:** Andrew S. Azman, John Rumunu, Abdinasir Abubakar, Haley West, Iza Ciglenecki, Trina Helderman, Joseph Francis Wamala, Olimpia de la Rosa Vázquez, William Perea, David A. Sack, Dominique Legros, Stephen Martin, Justin Lessler, Francisco J. Luquero

**Affiliations:** Johns Hopkins Bloomberg School of Public Health, Baltimore, Maryland, USA (A.S. Azman, D.A. Sack, J. Lessler, F.J. Luquero);; Ministry of Health, Juba, South Sudan (J. Rumunu);; World Health Organization, Juba (A. Abubakar, J.F. Wamala);; International Organization for Migration, Juba (H. West);; Médecins Sans Frontières, Geneva, Switzerland (I. Ciglenecki);; Medair, Ecublens, Switzerland (T. Helderman);; Médecins Sans Frontières, Barcelona, Spain (O. de la Rosa Vázquez);; World Health Organization, Geneva (W. Perea, D. Legros, S. Martin);; Epicentre, Paris, France (F.J. Luquero)

**Keywords:** cholera, vaccine, transmission dynamics, Vibrio cholerae, diarrhea, displaced persons, South Sudan, vulnerable populations, bacteria

## Abstract

Following mass population displacements in South Sudan, preventive cholera vaccination campaigns were conducted in displaced persons camps before a 2014 cholera outbreak. We compare cholera transmission in vaccinated and unvaccinated areas and show vaccination likely halted transmission within vaccinated areas, illustrating the potential for oral cholera vaccine to stop cholera transmission in vulnerable populations.

In December 2013, violence erupted in Juba, South Sudan, and quickly spread throughout the country. By the end of 2014, one in five persons within the country had been displaced, and many sought refuge in protection of civilians (PoC) sites inside United Nations (UN) Mission bases and in spontaneous internally displaced persons (IDP) settlements. Within 6 weeks of the start of the violence, South Sudan Ministry of Health requested vaccine from the global oral cholera vaccine stockpile to target 163,000 IDPs in 6 camps throughout the country, but not persons in the broader host communities (*1*).

In April 2014, two months after vaccine deployment, South Sudan confirmed the first case of cholera in the country since 2009; ≈4 weeks later, officials declared a cholera outbreak. Over 5 months, 6,269 suspected cholera cases were reported, including 156 deaths. Most cases occurred outside vaccinated camps, often in communities or camps surrounding vaccinated populations.

Several studies have demonstrated the individual-level (direct) effects of oral cholera vaccination (*2*–*4*), but few have estimated the overall population-level effect (a combination of direct and indirect effects), which is critical to determining costs and benefits. To estimate the overall effect, the observed epidemic in vaccinated areas must be compared with a counterfactual epidemic that is modeled or based on an observed suitable control population.

We used detailed epidemiologic data from the 2014 vaccination campaigns and the subsequent cholera outbreak in South Sudan to determine how vaccine use may have altered the epidemic course in vaccinated areas. We compared epidemics in 2 areas that included vaccinated and unvaccinated populations: 1) PoC sites (vaccinated) and the community (unvaccinated) in Juba; and 2) Malakal PoC (vaccinated) and Wau Shilluk IDP (unvaccinated), 2 similar camps separated by a river.

## The Study

The South Sudan Ministry of Health and World Health Organization implemented a clinic-based cholera surveillance system that captured basic patient data, laboratory results (if available), and outcomes. A suspected cholera case-patient was defined as anyone with acute watery diarrhea (diagnosed by a clinician); suspected cases were considered confirmed if the patient had a culture-positive fecal sample. Our analyses include all suspected cases.

We considered 5 populations in our comparisons, 3 in Juba County and 2 in Malakal County. In Juba, displaced persons were largely confined to 2 camps: 1) Tongping PoC camp (population 14,015) near the center of Juba; and 2) the UN House PoC camp (population 17,627) on the outskirts the city. We assumed all camp occupants were at risk for cholera and that, in the Juba community, only those residents without access to improved sanitation were at risk (*5*,*6*) (Technical Appendix).

Two-dose vaccine coverage among those eligible for vaccination (based on age and pregnancy status) was 93% in Tongping PoC and 95% at UN House; the remaining Juba population was not vaccinated (*1*). In Malakal, we compared an informal unvaccinated IDP settlement, Wau Shilluk (population 39,000; Technical Appendix), with an official PoC site, Malakal PoC camp (population 17,000; Technical Appendix). Two-dose vaccine coverage in Malakal was 92.2% based on a coverage survey using systematic random sampling (*1*).

We estimated the time-varying reproductive number of cholera within each location (Technical Appendix) (*7*). We assumed that the median generation time for cholera followed a gamma distribution with a median of 5 days and that all infectious cases were clinically apparent. We calculated 95% CIs by using a multiple imputation and bootstrapping routine, in which we first stochastically imputed missing or inconsistent symptom onset times and then resampled observations with replacement (Technical Appendix).

**Figure 1:**
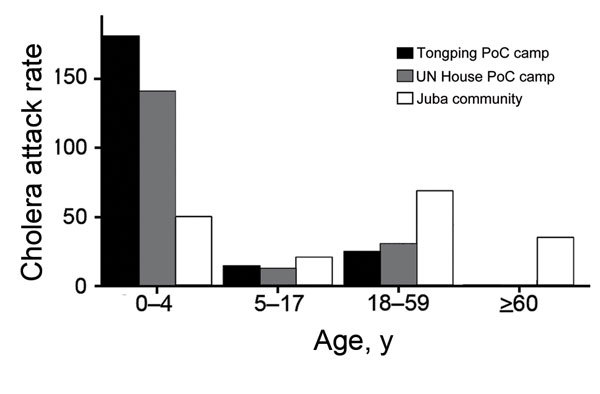
Estimated age-specific cholera attack rates (per 100,000 population) at different locations in Juba, South Sudan, 2014. PoC, protection of civilians; UN, United Nations.

The cholera attack rate in the Juba community was 53.4 cases/10,000 persons at risk (i.e., 2,229 cases/387,512 persons at risk), compared with 49.9 cases/10,000 persons at risk in the Juba camps (i.e., 158 cases/31,642 persons at risk). Although the overall attack rates were similar, the age distribution in camps differed markedly from those in the community. In the community, the risk for cholera among children <5 and those >5 years of age was nearly identical (risk ratio [RR] 1.0), but in the camps, the risk was substantially higher among children <5 years of age (Table; Figure 1). These age-specific differences in attack rates between camps and the community did not appear to be explained by population structure, age-specific vaccination coverage, or circulation of another diarrheal pathogen in the camps (Technical Appendix); the differences point toward possible lower vaccine effectiveness among young children. The response mounted to oral vaccines is weaker in children than adults (*8*), although considerable uncertainty remains regarding the response to the oral cholera vaccine. 

**Table:** Effect of oral cholera vaccine by location, South Sudan, 2014*

**Variable**	**Location**				
	Juba†	Tongping	UN House	Wau Shilluk†	Malakal
**Setting type**	Community	PoC Camp	PoC Camp	IDP camp	PoC camp
**Population vaccinated**	No	Yes	Yes	No	Yes
**Population at risk**	387,512	14,015	17,627	39,000	17,000
**No. cases/10,000 persons**	53.4	51.3	48.8	236.4	38.8
**No. cases/10,000 children <5 y of age **	56.0	186.5	146.5	–	–
**Risk ratio, children <5 y compared with those >5 y of age**	1.0	3.6	3.0	–	–
**No. days with R*_t_* >1**	16‡	2‡	2‡	14‡	2‡
**Maximum R*_t_***	2.4	1.5	1.5	2.2	1.9

*IDP, internally displaced person; PoC, protection of civilian; R*_t_*, reproductive number; UN, United Nations; –, no age-specific population data available. †Reference population. **‡Significant difference (p<0.0001) in number of days with R*_t_* >1, compared with reference population.**

The difference in the estimated cumulative cholera attack rates between the unvaccinated Wau Shilluk IDP camp (236.4 cases/10,000 persons at risk) and the vaccinated Malakal PoC camp (38.8 cases/10,000 persons at risk) was even more striking (incidence rate ratio 6.1) (Table). Age-specific population figures were unavailable for Wau Shilluk.

Although differences in attack rates suggest a likely reduction in cholera risk in vaccinated areas and the possibility of age-dependent vaccine protection, these estimates are uncertain and should be cautiously interpreted. An alternative approach to understanding the effect of vaccination is to compare observed cholera transmission dynamics within vaccinated and unvaccinated populations.

**Figure 2:**
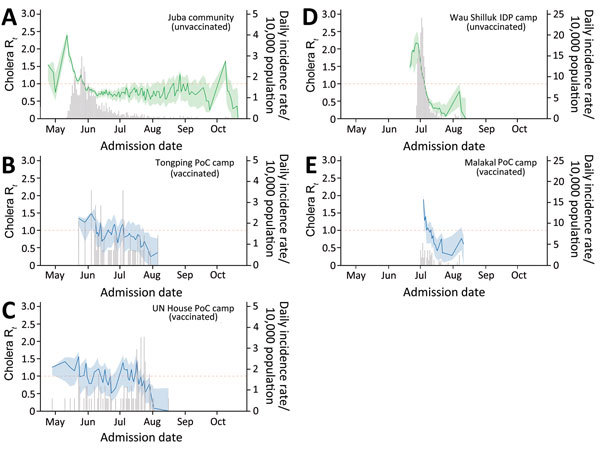
Estimates of the daily cholera reproductive number (R*_t_*) in vaccinated and unvaccinated populations, South Sudan, 2014. A–E) Bars indicate daily number of suspected cholera cases; green and blue lines represent the estimated R*_t_* in unvaccinated and vaccinated populations, respectively; and colored shading indicates 95% CIs for R*_t_* at Juba locations (A–C), Wau Shilluk IDP camp (D), and Malakal PoC camp. IDP, internally displaced persons; PoC, protection of civilians; UN, United Nations.

The epidemic curves within vaccinated camps in Juba had no distinct peak and suggest a series of cholera introductions with little to no onward transmission (Figure 2). We estimated that the daily reproductive number (R*_t_*; i.e., average number of secondary cases from a case becoming symptomatic on day *t*; Technical Appendix) in vaccinated camps was <1 for most of the epidemic. Each vaccinated camp had only 2 days on which the 95% CI of R*_t_* was above unity. This finding contrasts with our estimates in unvaccinated areas, where despite conditions that may have been less suitable for transmission, R*_t_* remained >1 for a sufficient and significantly longer time for an epidemic to progress (p<0.0001; Table; Technical Appendix).

## Conclusions

We show that cholera vaccination campaigns likely played a key role in curtailing cholera transmission in vaccinated areas within South Sudan. The age-specific transmission patterns within the vaccinated camps in Juba suggest that vaccinated young children were less protected in the camps, although further investigation is needed to explore this and other possible explanations, including age-specific differences in care-seeking behavior between populations.

Our study had several limitations. Analyses were based on suspected cases, which were defined by using a sensitive, but less specific, case definition; thus, many included cases were likely to be false positives. Our estimates of cholera attack rates depended on estimates of the population at risk in each area. We used the most reliable and up-to-date sources from agencies with an operational presence on the ground; however, the sizes of the dynamic community and camp populations used in the analyses were uncertain, and this uncertainty was not accounted for in the models. Last, we estimated the time-varying reproductive number of cholera by assuming a fixed generation time throughout the epidemic, which may not reflect reality due to the possibility of differences in care-seeking behavior and differential contraction of generation intervals between populations with an increasing prevalence of cholera (*9*).

Our findings provide evidence of the population-level effects of oral cholera vaccine. More work is needed to quantify this effect across multiple settings in reactive and preemptive deployments of the vaccine. High-quality surveillance and capacity to confirm suspect cases can greatly improve the possibility of making future estimates.

Technical Appendix Methods and technical details for estimates of cholera attack rates and daily cholera reproductive number and potential explanations for observed age distribution of suspected case-patients, South Sudan, 2014.
