# Supplementary material for: Population-Level Effect of Cholera Vaccine on Displaced Populations, South Sudan, 2014
Source: Emerg Infect Dis. 2016 Jun;22(6):1067–70. doi: 10.3201/eid2206.151592 (PMC4880069; doi:10.3201/eid2206.151592)
Supplement: Technical Appendix — Methods and technical details for estimates of cholera attack rates and daily cholera reproductive number and potential explanations for observed age distribution of suspected case-patients, South Sudan, 2014. [file 15-1592-Techapp-s1.pdf]

Article DOI: http://dx.doi.org/10.3201/eid2206.151592

Population-Level Effect of Cholera Vaccine on Displaced Populations, South Sudan, 2014

Technical Appendix

1. Estimation of Attack Rates

To estimate the attack rates in Juba 3/UN House (purple) and Tongping (orange) PoC sites, we had to first estimate the population at risk in the camps. To account for the dynamic population, we estimated the PoC site populations at the ‘case-weighted midpoint’ of the epidemic (Technical Appendix Figure 1). The population trajectory over time was estimated with a non-parametric spline model fit to camp population estimates at multiple time points from UN OCHA reports. We estimated the attack rate in Juba 3/UN House to be $10{,}000 \times \frac{86}{17{,}627} = 48.8$ per 10,000 and that of Tongping to be $10{,}000 \times \frac{72}{14{,}015} = 51.3$ per 10,000.

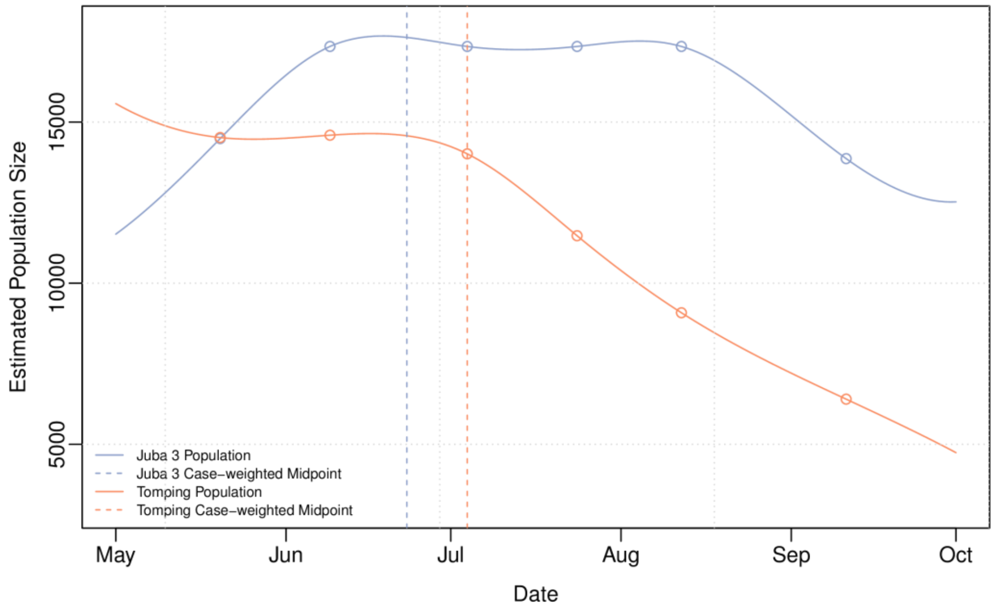

Technical Appendix Figure 1. Estimated population in Juba 3/UN House and Tongping PoC sites with the case-weighted epidemic midpoint noted as a dashed line. Data from UNOCHA reports shown as dots.

To estimate the population as risk in Juba county, we used UN OCHA data for the population from April 2014 (http://www.unocha.org/south-sudan/) and then subtracted the estimated (case-weighted) PoC site population. While it is clear the entire Juba population is not at risk for cholera, with the limited data available on population distribution and demographics within the city, it is difficult to estimate the true size of the at risk population. Following Ali et al (*1*), we assumed that only those without access to improved sanitation (likely to overlap with those who also have access to safe drinking water) as measured by the UNICEF/WHO Joint Monitoring Program (84%, http://www.wssinfo.org/) were at risk. This resulted in a final at risk population in Juba of 387,512. Thus, we estimated the attack rate to be $10{,}000 \times \frac{2{,}071}{387{,}512} = 53.4$ per 10,000. It is worth noting that if the entire population of Juba County (minus the camps in this calculation) were assumed to be at risk, the attack rate would then be $10{,}000 \times \frac{2{,}071}{461{,}324} = 44.9$ per 10,000, which is lower than that estimated in the camps.

Only a single point estimate for the population size, based on biometric registration data from July 2014, was available (from IOM) for the Malakal camp. Population data from Wau-Shilluk based on survey data from the same month was available based on use of the displacement tracking matrix methodology (http://southsudan.iom.int/wp-content/uploads/2014/08/DTM-Report-Round-IV.pdf and http://www.iomsouthsudan.org/tracking//dtm).

Age specific attack rates were estimated for the Juba locations. The age distribution for the Juba community was assumed to be equivalent as that for the entire country as estimated by the U.S. Census Bureau (http://www.census.gov/population/international/data/idb/informationGateway.php). A comparison of age distribution of suspected cholera attack rates in Juba community and camps to the estimated population structure is provided in Technical Appendix Figure 2.

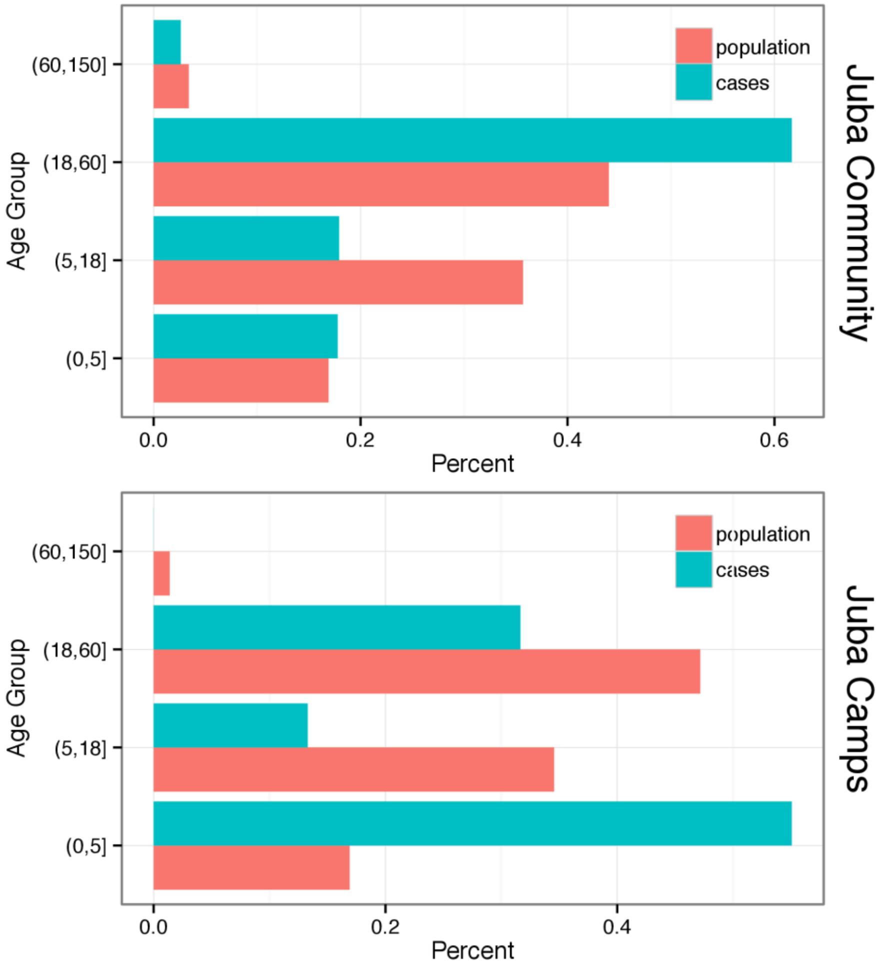

Technical Appendix Figure 2. Comparison of age distribution of suspected cholera attack rates in Juba community and camps to the estimated population structure.

2. Potential Explanations for the Observed Age Distribution of Suspected Cases in Juba Populations

In the Juba camps, we observed a far different age-distribution of suspected cholera cases than in the community (main text; Technical Appendix Figure 3). While, one possible explanation for this observation is lower vaccine effectiveness in children, in this section we briefly explore other potential explanations.

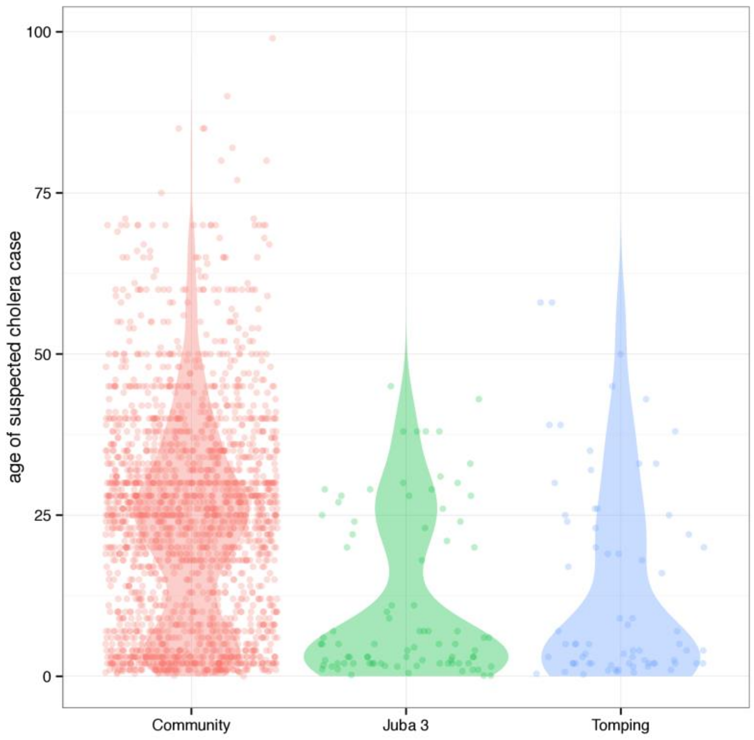

Technical Appendix Figure 3. Age distribution of suspected cases within the Juba community (red), Juba 3/UN House PoC site (green), and Tongping PoC site (blue). Dots represent each case and the colored polygon illustrates the distribution with wider areas representing a higher proportion of the cases of that age.

2.1 Differences in Historical Cholera Exposure

One possible explanation for the high attack rates in children in the Juba camps is that the IDPs came from a population with a different historical exposure pattern to cholera from people in the community. The median age of suspected cases in Upper Nile State (6 years old), one location where some IDPs came from, was significantly lower than that in Central Equatoria State (Camps and Community combined, 24 years old) (Technical Appendix Figure 4). If this observed age distribution was due to the immune landscape as opposed to differential care-seeking behavior, differences in suspected case definitions based on age or differences in the population structure, it could have contributed to the lower age of cases in the camps compared to the community in Juba. However, data collected in May 2014 based on camp registration data suggested that 85% of IDPs in Juba 3 and 96% of IDPs in Tongping came from Central Equatoria State (Camp Coordination Camp Management Cluster Displacement Tracking Matrix,

http://www.iomsouthsudan.org/tracking/). If this proportion were stable through the outbreak, it is unlikely that differences in historical cholera exposure could have driven our age-specific attack rate estimates in the Juba camps.

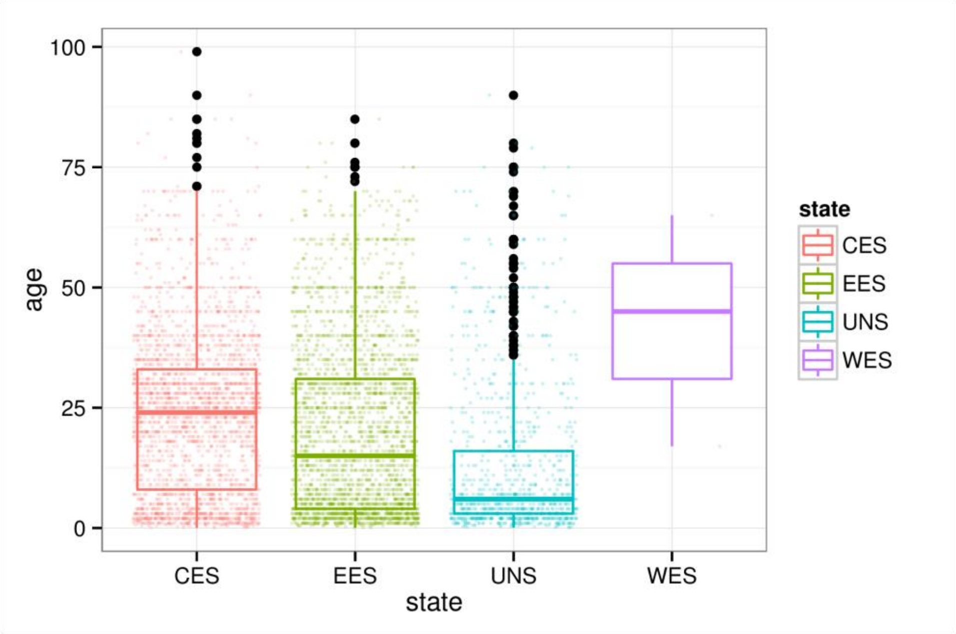

Technical Appendix Figure 4. Age distribution of suspected cases by State. CES = Central Equatoria, EES = Eastern Equatoria, UNS = Unity State and WES = Western Equatoria.

2.2 Possible Co-circulation of a Childhood Diarrheal Pathogen in the Camps

Exploring the proportion of rapid diagnostic test (RDT) positive suspected cases among those under-5 and those over-5 in the community in camps can provide us some additional insight into what may (or may not) have contributed to our estimates of high under-5 attack rates in the camps compared to the community. Among those tested with RDTs (Crystal VC, Span Diagnostics), we found that a higher proportion were cholera positive in the camps compared to the community (Technical Appendix Table), suggesting that the suspected case definition in the camps may have been more specific. We also see that the proportion of RDT-positive cases between under-5s and over-5s did not significantly differ within each setting (using 2-sample test of independent proportions as implemented in R using prop.test). This provides evidence against the hypothesis that another diarrheal pathogen circulated in the camps mostly among children leading to inflated suspected cases in children compared to adults in the camps. While interesting, these results should be interpreted with caution as it is unclear what the criteria were

for the use of RDT in the camps and the community, and this likely does not represent a true random sample of the suspected cases in each population.

Technical Appendix Table. Proportion of suspected cases tested who were rapid-test positive by population and age group.

Population	Under-5% Positive (n)	Over-5% Positive (n)
Community	0.28 (25)	0.27 (139)
Juba 3/UN House	0.89 (27)	0.78 (23)
Tongping	0.71(24)	0.74 (23)

2.3 Differences in Age-Specific Vaccine Coverage in Juba Camps

The LQAS survey referenced in the main text did not have a sufficient sample size to precisely estimate coverage by age within the Juba camps. However, they did collect age data on participants and they estimate that 100% of those under 5 received at least 1-dose and 80% received 2 doses in Tongping and UN House combined (n = 15). These are consistent with other OCV campaigns where coverage in young children has typically been high (*2*).

3. Estimation of R_t

We estimated the time-varying reproductive number using methods similar to that of Wallinga and Teunis (*3*). Since not all cases had a reported symptom onset date, we used the empirical distribution of the time from (self-reported) symptom onset to admission (Technical Appendix Figure 5) to impute the symptom onset dates for those individuals with missing or obviously inconsistent data (e.g., a symptom onset date after admission date).

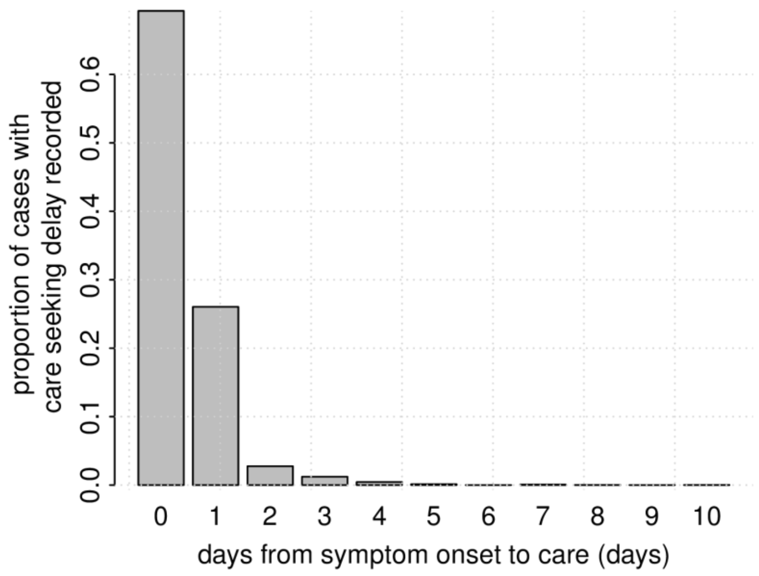

Technical Appendix Figure 5. Delay from self-reported symptom onset to admission for 5,222 suspected cases with data on both admission date and symptom onset date.

This method requires the use of a generation time distribution, or the distribution of the times between successive infections. While no estimates of generation time have been explicitly made, household data from Weil et al. (*4*) in Bangladesh point toward a mean generation time ranging from a few days up to ≈10 days. Consistent with previous publications (*5*), we assumed the median generation time was 5 days and further assumed it followed a gamma distribution, $\Gamma(rate = 0.1, shape = 0.5)$ (Technical Appendix Figure 6). Alternative distributions with similar medians were explored and led to qualitatively similar results.

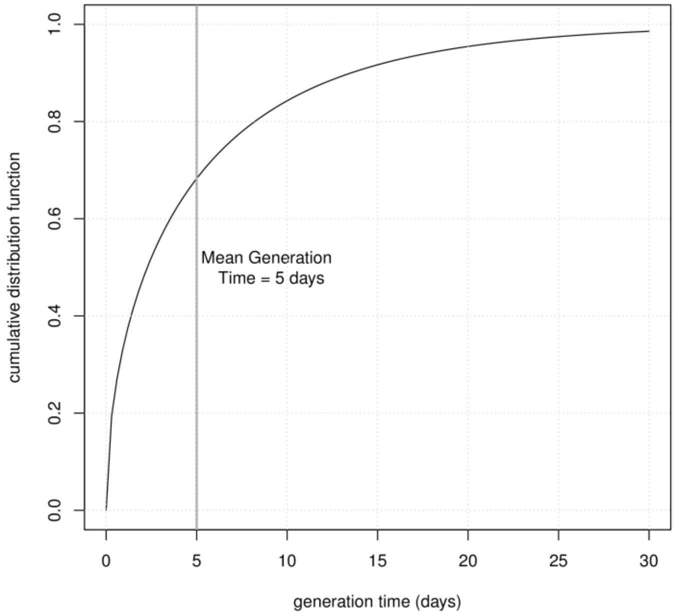

Technical Appendix Figure 6. Assumed distribution of generation time used to estimate R_t

By using this approach, we implicitly assume that all infectious cases are detected (i.e., asymptomatic cases and those not seeking care are not infectious). While there is evidence of mildly symptomatic and asymptotic infections occurring (*6*,*7*), they tend to shed orders of magnitude lower concentrations of bacteria and given that they are less symptomatic, they produce far less stool (*8*). Within these populations, it is likely that some infectious cases were missed by the surveillance system, though previous publications have shown that this method is relatively robust to cases being missing at random (i.e., the reporting probability for each person being less than 1) (*3*). If asymptomatic cases did contribute to secondary infections (at the same or different level of infectiousness as symptomatic cases) similarly in vaccinated and unvaccinated populations, we would expect our qualitative inference related to the impact of vaccination to remain intact.

We estimated the uncertainty in our estimates through an iterative bootstrapping routine where we first stochastically impute missing or inconsistent symptom onset times (e.g., a symptom onset date after admission date) and then resample with replacement 100 times. This routine was repeated 500 times and the 2.5th and 97.5th quantiles were used as the 95% confidence intervals.

To further support our findings that there were far fewer days (both as a number and as a proportion of epidemic days) where R_t>1, we used the bootstrap resamples to estimate the number of days for each location that R_t>1. We then tested the differences between comparison areas with a Wilcoxan Rank Sum test, with a null hypothesis that the number of days with R_t>1 was the same in each population. As we might expect more days in larger populations (like Juba compared to the camps), we also treated each bootstrap as a binomial observation to test whether the proportion of days with R_t>1 differed between the two populations using a simple logistic regression model where the dependent variable was an indicator for R_t>1(one observation for each day in each location) and the dependent variable was location (performed separately for the two comparison groups). As reported in the main text, we found that the probability of any day having a reproductive number greater than unity was significantly larger in unvaccinated camps than vaccinated camps.

References

1. Ali M, Lopez AL, You YA, Kim YE, Sah B, Maskery B, et al. The global burden of cholera. Bull World Health Organ. 2012;90:209–218A. PubMed http://dx.doi.org/10.2471/BLT.11.093427

2. Luquero FJ, Grout L, Ciglenecki I, Sakoba K, Traore B, Heile M, et al. First outbreak response using an oral cholera vaccine in Africa: vaccine coverage, acceptability and surveillance of adverse events, Guinea, 2012. PLoS Negl Trop Dis. 2013;7:e2465. PubMed http://dx.doi.org/10.1371/journal.pntd.0002465

3. Wallinga J, Teunis P. Different epidemic curves for severe acute respiratory syndrome reveal similar impacts of control measures. Am J Epidemiol. 2004;160:509–16. PubMed http://dx.doi.org/10.1093/aje/kwh255

4. Weil AA, Khan AI, Chowdhury F, LaRocque RC, Faruque ASG, Ryan ET, et al. Clinical outcomes in household contacts of patients with cholera in Bangladesh. Clin Infect Dis. 2009;49:1473–9. PubMed http://dx.doi.org/10.1086/644779

5. Azman AS, Luquero FJ, Rodrigues A, Palma PP, Grais RF, Banga CN, et al. Urban cholera transmission hotspots and their implications for reactive vaccination: evidence from Bissau city, Guinea Bissau. PLoS Negl Trop Dis. 2012;6:e1901. PubMed http://dx.doi.org/10.1371/journal.pntd.0001901

6. Jackson BR, Talkington DF, Pruckler JM, Fouché MDB, Lafosse E, Nygren B, et al. Seroepidemiologic survey of epidemic cholera in Haiti to assess spectrum of illness and risk factors for severe disease. Am J Trop Med Hyg. 2013;89:654–64. PubMed http://dx.doi.org/10.4269/ajtmh.13-0208

7. Clements ML, Levine MM, Young CR. Magnitude, kinetics, and duration of vibriocidal antibody responses in North Americans after ingestion of *Vibrio cholerae*. J Infect Dis. 1982;145:465–73. PubMed http://dx.doi.org/10.1093/infdis/145.4.465

8. Black RE, Levine MM, Clements ML, Young CR, Svennerholm AM, Holmgren J. Protective efficacy in humans of killed whole-vibrio oral cholera vaccine with and without the B subunit of cholera toxin. Infect Immun. 1987;55:1116–20. PubMed
